# Editorial: Role of epigenetics in environmental pollution associated diseases

**DOI:** 10.3389/fgene.2023.1266714

**Published:** 2023-08-01

**Authors:** Xiang He, Jiliu Liu, Guoping Li

**Affiliations:** ^1^ Laboratory of Allergy and Precision Medicine, Chengdu Institute of Respiratory Health, The Third People’s Hospital of Chengdu, Affiliated Hospital of Southwest Jiaotong University, Chengdu, China; ^2^ Department of Pulmonary and Critical Care Medicine, Chengdu Third People’s Hospital Branch of National Clinical Research Center for Respiratory Disease, Affiliated Hospital of Chongqing Medical University, Chengdu, China

**Keywords:** environmental pollution, DNA methylation, RNA methylation, cancers, IPF, biomarker

The understanding of the pathogenesis and associated molecules of various diseases remains limited, and the targets for prevention and treatment have not been fully elucidated. Genetic and epigenetic modifications, including DNA methylation, RNA methylation, and histone modifications, can also impact the phenotype of many diseases. Environmental pollutants, such as airborne pollutants (e.g., heavy metals and fine particulate matter) and ingested pollutants (e.g., cigarettes), have become significant contributors to diseases in today’s world.

In recent years, there has been increased attention from researchers on the relationship between gene epigenetic modifications and environmental exposures. Studies have shown that many environmental exposures have a considerable impact on gene epigenetic modifications, which can persist for years after the exposures have ceased. Therefore, understanding the relationship between environmental factors and epigenetics can help uncover key factors in disease development and facilitate early prevention and treatment. The focus of this Research Topic is to identify environmental factors that epidemiologically influence disease susceptibility, explore the molecular-level relationship between exposure to different environmental pollutants and gene epigenetic modifications, elucidate their molecular regulatory mechanisms, and investigate the interrelationships between various stages of disease development and relevant environmental exposures along with their dynamics. These efforts can help identify targets for disease prevention and treatment.

To investigate the role of N6-methyladenosine (m6A) modification in colorectal cancer (CRC) recurrence and its impact on the tumor microenvironment (TME), Zhu et al. evaluated the m6A modification patterns of 804 primary CRC patients using 27 m6A regulators. They identified distinct patterns that influence immune cell infiltration in the TME and have implications for prognosis. Six genes, TOP2A, LRRC58, HAUS6, SMC4, ACVRL1, and KPNB1, were associated with CRC recurrence. This research sheds light on the importance of m6A modification in CRC recurrence and provides insights into TME mechanisms.

Zhu et al. identified the key gene STC2, regulated by PM2.5 exposure, across multiple cancers using pan-cancer information. STC2 expression was associated with different prognoses in various cancers and correlated with clinical factors. STC2 also positively correlated with RNA methylation genes and immunomodulators across tumors. The study highlights the impact of PM2.5 on cancer development and the clinical significance of STC2 in air pollution-related cancers.

Zhang et al. assessed m6A modification patterns in patients with idiopathic pulmonary fibrosis (IPF). They identified specific m6A modulators with diagnostic potential and found that METTL14 and G3BP2 could classify IPF patients into different risk groups based on m6A modification patterns. m6A modification may stabilize mRNA associated with tight junctions, and upregulated neutrophil expression in high-risk m6A groups indicated poor outcomes. The researchers also evaluated drug sensitivity based on m6A modification patterns.

For DNA epigenetic modifications, Zhao et al. investigated the relationship between environmental chromium (Cr) exposure and DNA methylation. They identified significant CpG sites corresponding to four genes, including a differentially methylated region (DMR) associated with the ALDH3A1 gene involved in cell proliferation and tumor promotion. Future studies with larger sample sizes are needed to validate these findings and establish a comprehensive understanding of the epigenetic effects of environmental Cr exposure.

Overall, we expect this Research Topic to significantly contribute to understanding epigenetic alterations induced by environmental pollutants in disease development, offering insights for effective prevention and treatment. By delving deeper into the intricate relationship between environmental factors and epigenetics, we aim to unravel crucial mechanisms underlying disease pathogenesis and progression, thereby paving the way for early intervention and targeted treatments. By shedding light on the complex interplay between environmental pollutants and epigenetic modifications, this research has the potential to revolutionize our approach to disease management.

